# Cyclic nucleotide‐dependent inhibitory signaling interweaves with activating pathways to determine platelet responses

**DOI:** 10.1002/rth2.12122

**Published:** 2018-06-12

**Authors:** Zoltan Nagy, Albert Smolenski

**Affiliations:** ^1^ Institute of Cardiovascular Sciences College of Medical and Dental Sciences University of Birmingham Birmingham UK; ^2^ UCD School of Medicine University College Dublin Dublin Ireland; ^3^ UCD Conway Institute University College Dublin Dublin Ireland; ^4^ Irish Centre for Vascular Biology Royal College of Surgeons in Ireland Dublin Ireland

**Keywords:** 14‐3‐3 protein, cyclic AMP, G‐protein, kinase, protein kinase A, RGS18

## Abstract

Platelets are regulated by extracellular cues that impact on intracellular signaling. The endothelium releases prostacyclin and nitric oxide which stimulate the synthesis of cyclic nucleotides cAMP and cGMP leading to platelet inhibition. Other inhibitory mechanisms involve immunoreceptor tyrosine‐based inhibition motif‐containing receptors, intracellular receptors and receptor desensitization. Inhibitory cyclic nucleotide pathways are traditionally thought to represent a passive background system keeping platelets in a quiescent state. In contrast, cyclic nucleotides are increasingly seen to be dynamically involved in most aspects of platelet regulation. This review focuses on crosstalk between activating and cyclic nucleotide‐mediated inhibitory pathways highlighting emerging new hub structures and signaling mechanisms. In particular, interactions of plasma membrane receptors like P2Y12 and GPIb/IX/V with the cyclic nucleotide system are described. Furthermore, differential regulation of the RGS18 complex, second messengers, protein kinases, and phosphatases are presented, and control over small G‐proteins by guanine‐nucleotide exchange factors and GTPase‐activating proteins are outlined. Possible clinical implications of signaling crosstalk are discussed.

**Essentials**

- Cyclic nucleotide‐dependent inhibition and platelet activation are depicted as separate pathways.
- Emerging evidence suggests that these pathways are intertwined at multiple levels.
- Platelet inhibition and activation often regulate the same signaling nodes in opposite directions.
- Better understanding of cross‐inhibition between these pathways will advance antiplatelet therapy.

## 1 INTRODUCTION

**Figure 1:**
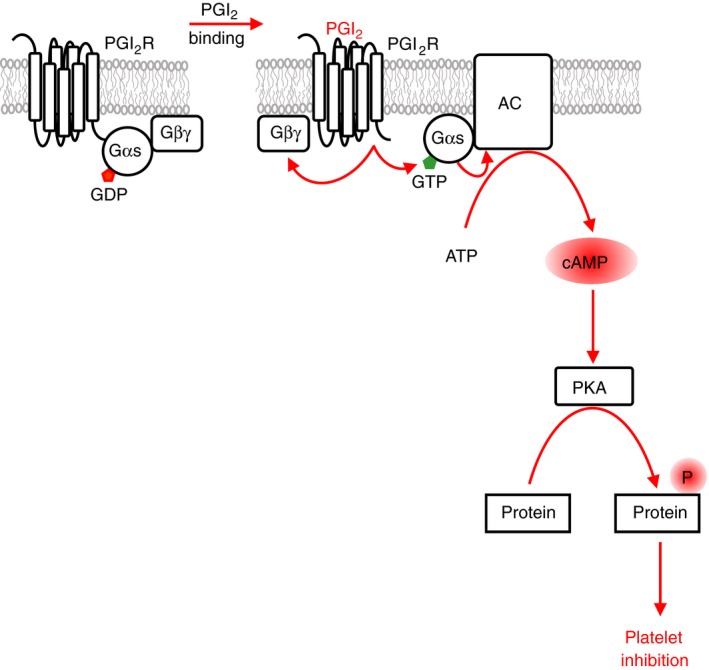
Prostacyclin signaling. Prostacyclin (PGI_2_) is released by healthy endothelial cells. The prostacyclin receptor on platelets couples to the stimulatory heterotrimeric Gs protein complex. Prostacylin binding leads to a conformational change in the receptor activating its guanine‐nucleotide exchange factor (GEF) activity towards Gαs resulting in the exchange of GDP by GTP. Gαs‐GTP binds to and activates the transmembrane protein adenylate cyclase (AC) to synthesize cAMP from ATP. The second messenger cAMP has only one major target in platelets which is the cAMP‐dependent protein kinase (PKA) family. cAMP binding to the regulatory subunits of PKA leads to activation of the catalytic subunits and to the phosphorylation of numerous substrate proteins resulting in a profound inhibition of most platelet functions

**Figure 2:**
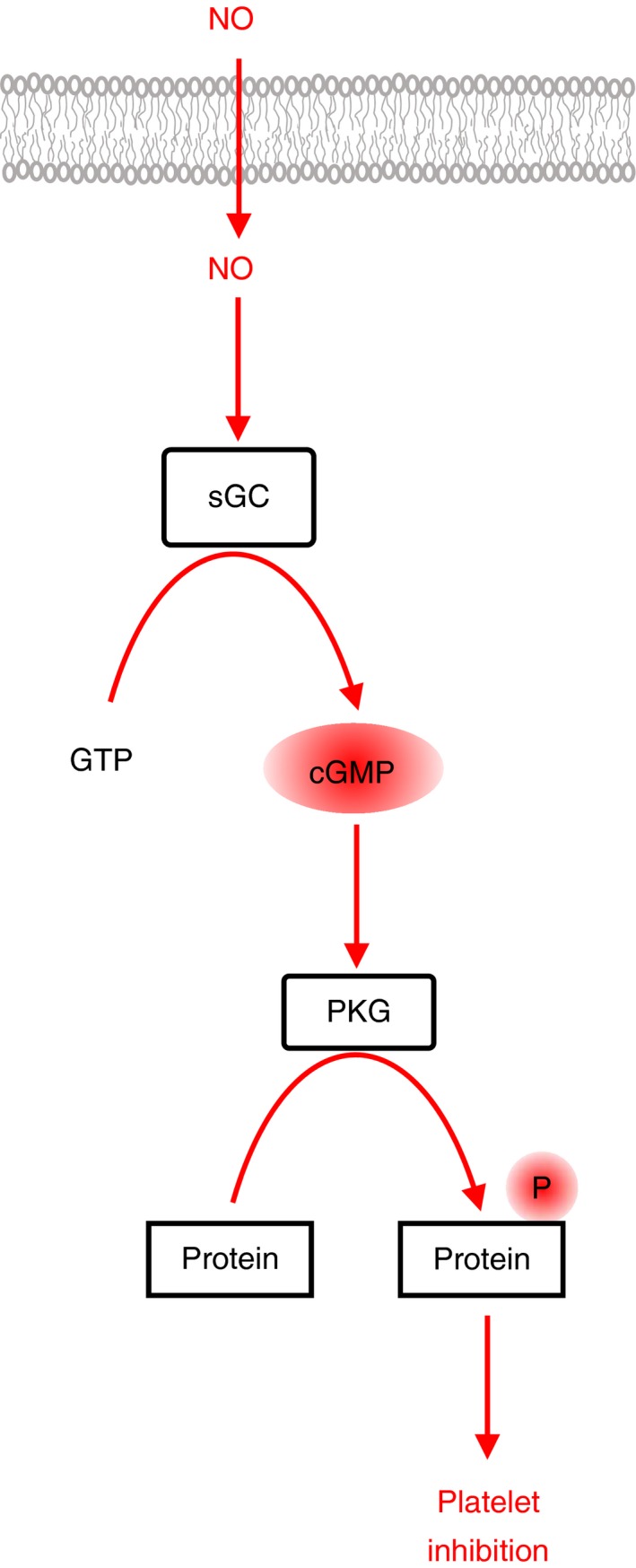
Nitric oxide signaling. Nitric oxide (NO) is released by healthy endothelial cells. NO is a small gaseous molecule that diffuses through the plasma membrane of platelets. The main target of NO in platelets is the soluble guanylate cyclase (sGC) which is stimulated by NO binding to synthesize the second messenger cGMP from GTP. The sole target of cGMP in platelets is the Iβ isoform of cGMP‐dependent protein kinase (PKG). cGMP binding to PKG Iβ activates the kinase domain to phosphorylate many substrate proteins resulting in a profound inhibition of most platelet functions

Platelets play a key role in vascular homeostasis. Platelet functions are tightly controlled by signaling pathways that respond to diverse extracellular cues. Healthy endothelium releases short‐lived mediators prostacyclin (PGI_2_) and nitric oxide (NO). PGI_2_ binds to a G‐protein coupled receptor (GPCR) which is linked to the Gs heterotrimeric G‐protein that stimulates cAMP synthesis by adenylate cyclase (AC). cAMP binds to the regulatory domains of cAMP‐dependent protein kinase (isoforms PKA I and II) leading to activation of the PKA catalytic subunits and to phosphorylation of a multitude of substrate proteins (Figure 1).^1^ NO diffuses through the plasma membrane and activates guanylate cyclase (sGC, NO‐GC) directly to produce cGMP. cGMP activates cGMP‐dependent protein kinase (isoform PKG Iβ) which phosphorylates substrate proteins that are often identical to the ones phosphoryated by PKA (Figure 2).^1^, ^2^ Cytosolic levels of the cyclic nucleotides are further controlled by phosphodiesterases (PDE) that degrade cAMP and cGMP. The signaling pathways induced by PGI_2_ and NO are thought to keep platelets in a quiescent state in the absence of damage.

In contrast, exposure of the extracellular matrix protein collagen and binding of von Willebrand factor (VWF) after vascular injury initiates platelet activation. Collagen stimulates the immunoreceptor tyrosine‐based activation motif (ITAM)‐containing glycoprotein VI (GPVI)‐Fc receptor γ chain complex which signals via Src family and Syk tyrosine kinases, whereas VWF binds to the GPIb/IX/V complex. In parallel, vascular damage also triggers the clotting cascade leading to thrombin generation which directly activates platelets via thrombin receptors and converts soluble fibrinogen into a fibrin mesh. Activated platelets release ADP and thromboxane A2 (TxA_2_) which bind to specific GPCRs. The signaling events downstream of these receptors include the activation of diverse kinases and GPCR‐linked heterotrimeric G‐proteins as well as small G‐proteins of the Ras and Rho families. Furthermore, calcium ions (Ca^2+^) are released from intracellular stores leading to further signal amplification ultimately inducing membrane and cytoskeletal reorganization, granule release and integrin activation.^3^, ^4^, ^5^ In addition to endothelium‐dependent PGI_2_ and NO pathways, platelet activation is limited by negative feedback through immunoreceptor tyrosine‐based inhibition motif (ITIM)‐containing receptors G6b‐B and PECAM‐1,^6^ serine/threonine phosphatases, intracellular receptors and mechanisms of receptor desensitization and cleavage.^5^ The ectonucleotidase CD39 expressed by endothelial cells hydrolyses ATP and ADP, thus limiting the availability of platelet agonists. The inhibitory functions of these molecules are often deduced from the phenotypes of the corresponding knockout mouse models (ie, hyper‐reactive platelets).

Cyclic nucleotide‐mediated inhibition and platelet activation are commonly described as separate systems. However, increasing evidence suggests an alternative model whereby simultaneous regulation of both systems determines platelet function. For example, to prevent platelet activation, inhibitory signaling targets key nodes in activating pathways.^7^ On the other hand, platelet activation requires active blockage of endothelium‐dependent inhibitory pathways,^8^, ^9^, ^10^, ^11^ including ADP‐mediated inhibition of cAMP production via P2Y12 and thrombin‐mediated cAMP degradation via PDE type 3A. Interestingly, both P2Y12 and PDE3A are targets of multiple clinically used drugs which reduce thrombosis.^12^ Once initiated, cyclic nucleotides are also able to reverse platelet aggregation leading to complete disaggregation of already formed platelet aggregates.^13^, ^14^, ^15^, ^16^ Given the powerful activity of endothelium‐derived inhibitors combined with the relative abundance of endothelial cells compared to platelets,^17^ cyclic nucleotide pathways are likely to contribute more significantly to the control of platelet activation than is usually appreciated. Recent comprehensive phosphoproteome studies indicate an increasing complexity in signaling events following binding of single agonists or combinations of agonists to platelet receptors activating each of these pathways.^18^, ^19^

In this paper, we focus on the interdependence of cyclic nucleotide‐dependent inhibitory and platelet activation pathways. To modulate each other's actions, these pathways constantly interact at multiple levels. We will describe these interactions highlighting recently identified protein‐protein assemblies involved in platelet control like the RGS18 complex. The potential clinical relevance of crosstalk between endothelium‐dependent inhibition and platelet activation will also be considered.

## 2 INTERACTIONS AT RECEPTOR LEVEL

### 2.1 Gi signaling

**Figure 3:**
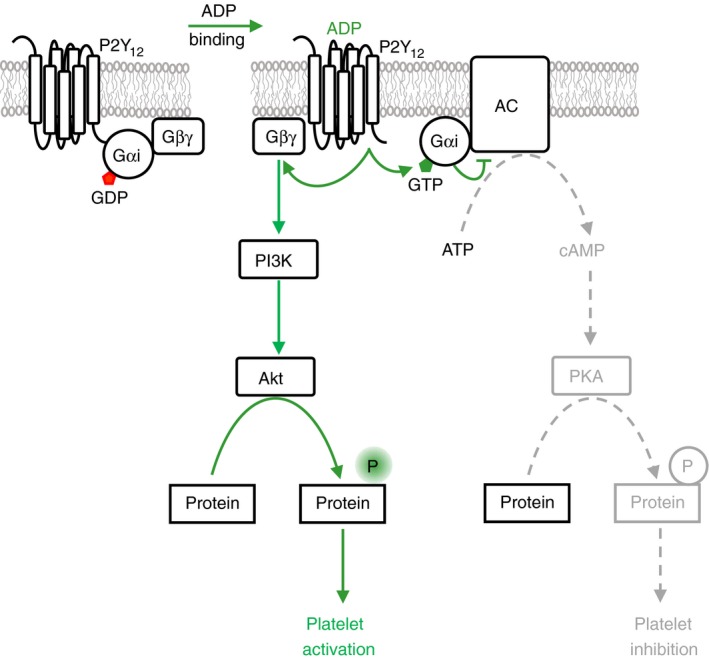
Gi signaling. The P2Y12 receptor for ADP is coupled to an inhibitory heterotrimeric Gi protein complex composed of Gαi and Gβγ subunits. ADP binding leads to exchange of GDP by GTP on the Gαi subunit and to the dissocation of the Gβγ subunit. The Gαi‐GTP subunit binds to and inhibits the function of adenylate cyclase (AC) resulting in a reduction of prostacyclin and cAMP/PKA signaling and diminished platelet inhibition. The Gβγ subunit binds to and activates phosphatidylinositol 3‐kinase β and γ (PI3K) to synthesize the membrane lipid phosphatidylinositol 3,4,5‐trisphosphate (PIP_3_) from phosphatidylinositol 4,5‐bisphosphate. PIP_3_ serves as docking site to recruit and activate the kinase Akt which phosphorylates substrate proteins contributing to platelet activation

The plasma membrane contains multiple receptor and regulatory proteins required for the initiation of signaling events leading to platelet inhibition and/or activation. Major receptors for platelet activators like ADP, epinephrine, and prostaglandin E2 (PGE2) work, at least in part, by inhibiting cAMP production, and thus by interfering with inhibitory pathways. Binding of ADP to the P2Y12 receptor causes a conformational change in the receptor allowing it to act as a guanine‐nucleotide exchange factor (GEF) and activate the membrane‐associated heterotrimeric G‐protein of the Gi‐family. The active GTP‐bound Gαi‐subunit dissociates from the β and γ subunits and binds to AC leading to diminished cAMP synthesis (Figure 3 and Table 1). Simultaneously Gi triggers the phosphatidylinositol 3‐kinase (PI3K) pathway, which converts phosphatidylinositol 4,5‐bisphosphate to phosphatidylinositol 3,4,5‐trisphosphate at the plasma membrane leading to the activation of the kinase Akt and inhibition of the Rap1‐GAP RASA3, key activating pathways in platelets.^20^, ^21^, ^22^ The synergism between P2Y12 function and blockage of cyclic nucleotide pathways has been shown for both, cAMP and cGMP components, although cGMP regulation by P2Y12 is not well understood.^9^, ^23^ These studies indicate that P2Y12‐mediated inhibition of cyclic nucleotide signaling is mandatory to achieve full platelet activation. Accordingly, knockout of the P2Y12 receptor in mice, congenital deficiency of the receptor in humans, or expression of constitutively active Gαi2 in a mouse model confirm the importance of P2Y12 signaling for platelet aggregation and thrombus formation.^10^, ^24^, ^25^ In thrombosis studies, the establishment of a tight vascular seal has been suggested to depend on P2Y12 function,^26^ and thus on cross‐inhibition of cAMP/cGMP pathways. P2Y12 and Gi signaling also contribute to the formation of the outer shell structure of hemostatic thrombi.^27^

**Table 1:** Examples of proteins differentially regulated by platelet activators and cyclic nucleotide‐dependent inhibitors

Protein	Effect of platelet activators	Effect of cyclic nucleotide‐dependent inhibitors
Adenylate cyclase	Gi mediated inhibition (induced by P2Y12,^23^ α2A‐adrenergic, and EP3 receptors^28^)	Gs mediated activation (prostacyclin receptor)
Soluble guanylate cyclase	Thrombospondin‐induced inhibition,^63^ VWF‐induced partial activation^51^	NO‐induced maximal activation^50^
RGS18	Activation and reduction of Gq signaling,^35^ inhibition leading to increased Ca^2+^ levels^7^	Activation leading to reduced Ca^2+^ levels^7^, ^37^
GPIbα receptor	Receptor shedding	Inhibition of shedding^46^
GPVI receptor	Dimerization	Inhibition of dimerization^48^
IP_3_‐receptor	Opening and Ca^2+^‐release	Inhibition^113^
PDE3A	Activation leading to reduced cAMP levels^11^	Inhibition by cGMP leading to elevated cAMP levels,^73^ activation by cAMP^71^
ERK	Activation	Inhibition^78^
p38 MAPK	Activation	Inhibition^78^
Rap1B	Activation	Inhibition^114^
Rap1GAP2	Inhibition leading to enhanced cell adhesion^92^	Activation leading to reduced adhesion^92^
Rac1	Activation^95^	Inhibition^94^, ^95^
RhoA	Activation^95^	Inhibition^95^
Arf6	Inhibition^98^	Activation^98^
Myosin light chain	Increased phosphorylation	Decreased phosphorylation^76^

This type of crosstalk is not restricted to the P2Y12 receptor alone. The α2A‐adrenergic and EP3 receptors for epinephrine and PGE2, respectively, are also coupled to Gi‐family proteins. PGE2 can be released from activated platelets providing positive feedback on platelet activation at least in part through inhibition of cAMP synthesis.^28^ Mathematical modelling suggests that the EP3 receptor might be even more potent than the P2Y12 receptor in inhibiting cAMP production.^29^ Another example of a Gi‐coupled receptor in platelets is the chemokine receptor CXCR4.^30^, ^31^ The importance of costimulatory Gi signaling was recently highlighted in a study of matrix metalloproteinase 2 which stimulates protease‐activated receptor PAR1 without being able to induce platelet aggregation in itself because of a lack of concomitant Gi activation.^32^

### 2.2 RGS18 complex

**Figure 4:**
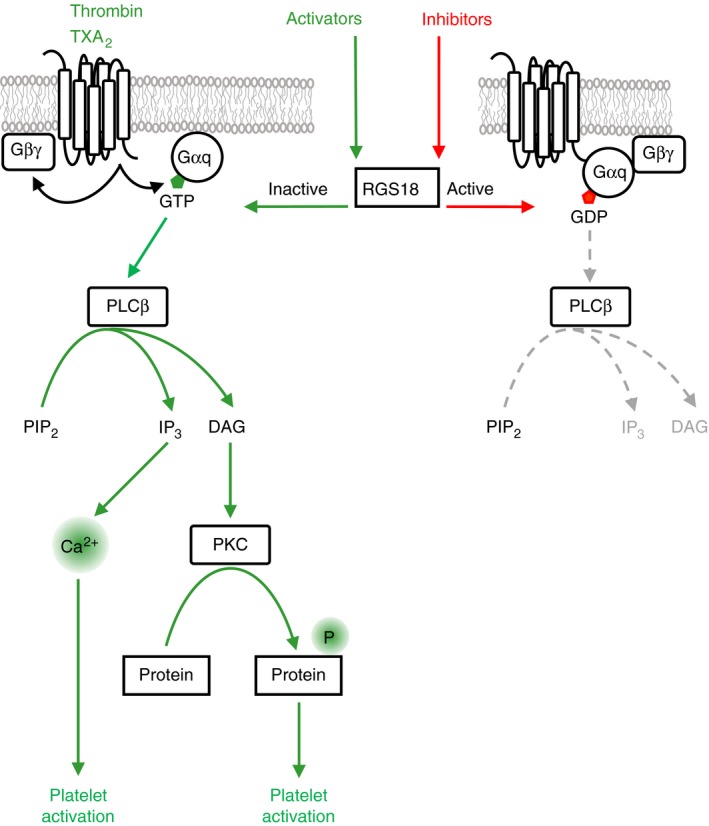
Regulation of Gq signaling by RGS18. Binding of the platelet activators thrombin and thromboxane A2 (TXA_2_) to their respective receptors induces the formation of active Gαq‐GTP which binds to and activates phospholipase Cβ (PLCβ) leading to the generation of inositol‐3‐phosphate (IP_3_) and diacylglycerol (DAG) from phosphatidylinositol 4,5‐bisphosphate (PIP_2_). IP_3_ triggers the release of calcium ions (Ca^2+^) from intracellular stores which are essential for platelet activation. DAG activates protein kinase C (PKC) isoforms which phosphorylate many proteins supporting platelet activation. Regulator of G‐protein signaling 18 (RGS18) is a GTPase‐activating protein (GAP) which accelerates the intrinsic GTPase activity of Gαq. RGS18 enables the hydrolysis of GTP bound to Gαq resulting in the formation of inactive Gαq‐GDP and in the termination of Gq signaling. Platelet activators like thrombin and TXA_2_ inhibit RGS18 thus support Gq signaling, whereas platelet inhibitors stimulate RGS18 function to stop Gq signaling

**Figure 5:**
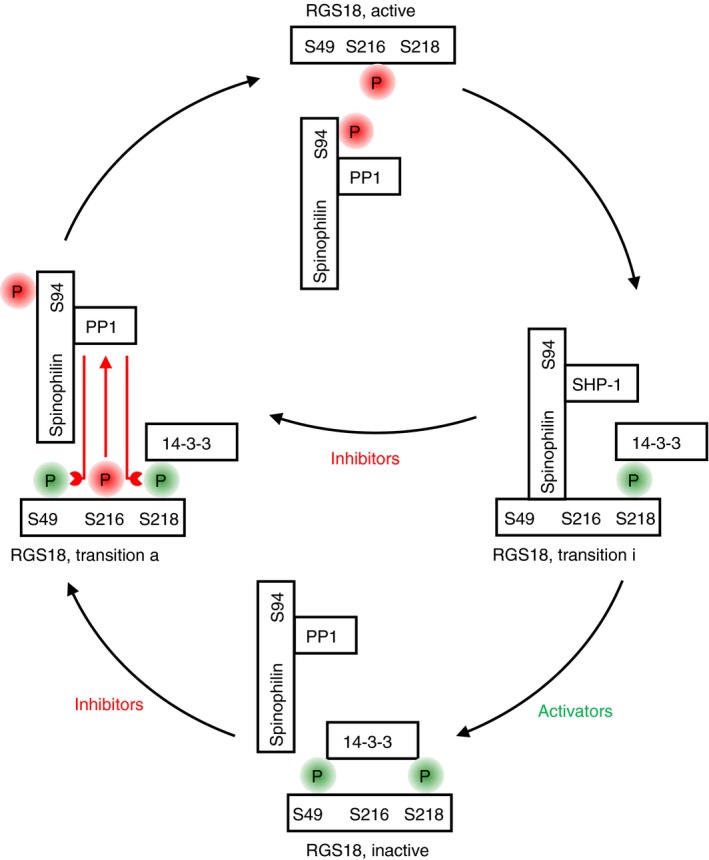
Activation/inactivation cycle of RGS18. Regulator of G‐protein signaling 18 (RGS18) terminates Gq signaling and is regulated by both platelet activating and inhibitory pathways. In freshly isolated resting platelets RGS18 is found in a complex with the adapter protein spinophilin, the tyrosine phosphatase SHP‐1 and the phospho‐serine/threonine binding protein 14‐3‐3γ attached to phosphorylated serine 218 of RGS18 (transition state on the way towards inactivation, transition i). Platelet activators like thrombin and TXA_2_ induce the phosphorylation of serine 49 of RGS18 generating a second 14‐3‐3 binding site leading to enhanced 14‐3‐3 binding. Simultaneously, SHP‐1 is activated and detaches from spinophilin (involving de‐phosphorylation of tyrosine residues on spinophilin), the serine/threonine phosphatase PP1 binds to spinophilin instead, and spinophilin dissociates from RGS18. In this state the RGS18 complex is inactive. Platelet inhibitors like prostacylin and nitric oxide induce the phosphorylation of serine 216 on RGS18 and serine 94 on spinophilin which lead to the activation of PP1 and detachment of spinophilin from RGS18 (transition state on the way towards activation, transition a). Active PP1 removes phosphate groups from serines 49 and 218 of RGS18 leading to the loss of 14‐3‐3 binding. This state of the RGS18 complex is characterized by free catalytically active RGS18 which can hydrolyse Gαq‐GTP to form inactive Gαq‐GDP (Figure 4). Phosphorylation sites linked to platelet inhibition are highlighted in red, whereas sites linked to platelet activation are marked in green

GPCR signaling is terminated by hydrolysis of GTP bound to Gα‐subunits of heterotrimeric G‐proteins. GTP hydrolysis is enabled by a specific family of GTPase‐activating proteins (GAPs) called regulators of G‐protein signaling (RGS) which accelerate the intrinsic GTPase activity of Gα subunits. RGS18 is a highly expressed GAP of Gαi and Gαq in platelets (Figure 4). Deletion of RGS18 in mice results in platelet activation marked by enhanced thrombus formation, platelet granule release and aggregation particularly in response to thrombin and TxA_2_.^33^, ^34^ RGS18 and its associated proteins have emerged as a highly regulated hub structure that coordinates cyclic nucleotide‐mediated inhibitory signaling and platelet activation. In resting platelets, RGS18 interacts with the scaffold protein spinophilin (neurabin‐2, PPP1R9B), the tyrosine phosphatase SHP‐1, and the phospho‐serine/threonine binding adapter protein 14‐3‐3 (Figure 5, RGS18 complex in transition i). Platelet activation by thrombin and TxA_2_ induces a dissociation of RGS18 and SHP‐1 from spinophilin^35^ and increases binding of 14‐3‐3γ to RGS18.^7^ 14‐3‐3 bound RGS18 is less active leading to enhanced Gq signaling and Ca^2+^‐release (Figure 5, RGS18 complex inactive). Loss of SHP‐1 during platelet activation leads to binding of the serine/threonine phosphatase PP1 to spinophilin which is associated with reduced PP1 activity towards myosin light chain (MLC) potentially contributing to enhanced MLC phosphorylation and platelet activation.^36^ Cyclic nucleotide inhibitory pathways also disrupt the RGS18/spinophilin/SHP‐1 complex,^37^ however, they inhibit binding of 14‐3‐3γ to RGS18 (Figure 5, RGS18 complex active).^7^ This free RGS18 binds more effectively to Gαi2 and attenuates Gq signaling leading to reduced Ca^2+^‐release and thus contributing to platelet inhibition.^7^, ^37^ The reorganization of RGS18 complexes is mediated by multiple de‐/phosphorylation events. Thrombin and TxA_2_ induce SHP‐1 Y536 phosphorylation leading to phosphatase activation and de‐phosphorylation of tyrosines on spinophilin thus contributing to dissolution of the spinophilin/SHP‐1/RGS18 complex.^37^ The role of SHP‐1 phosphatase activity in inducing complex dissociation is supported by pharmacological studies using a nonselective SHP‐1/SHP‐2 inhibitor (NSC‐87877).^35^ Thrombin, TxA_2_, and ADP induce phosphorylation of RGS18 on S49 resulting in enhanced 14‐3‐3 binding to RGS18.^38^ In contrast, PKA phosphorylates S216 of RGS18 leading to de‐phosphorylation of S49 and S218, presumably by PP1 (Figure 5, RGS18 complex in transition a), and to detachment of 14‐3‐3 from RGS18.^38^ The PKA‐induced release of RGS18 from spinophilin is supported by phosphorylation of spinophilin on S94.^37^

Interestingly, spinophilin knockout platelets exhibit reduced Ca^2+^‐release and aggregation which could, at least in part, be due to dysregulated RGS18 function.^35^ Absence of spinophilin also results in reduced cAMP synthesis in response to PGI_2_, highlighting multiple functions of this scaffold protein in platelets. Studies on SHP‐1‐deficient mice have identified a main role of SHP‐1 in regulating GPVI and integrin αIIbß3 signaling but did not reveal any defect in GPCR signaling.^39^ Because SHP‐1 is ablated during megakaryocyte differentiation in these mice, it is possible that the role of SHP‐1 in regulating GPCR signaling is not apparent because of compensatory mechanisms and/or redundancy with the closely related SHP‐2 phosphatase. Indeed, platelets deficient in both SHP‐1 and SHP‐2, display marked defects in PAR4 receptor signaling, suggesting a level of redundancy.^39^ Whether a spinophilin/RGS18 complex exists in SHP‐1 deficient platelets remains to be determined.

### 2.3 GPIb/IX/V complex

The GPIb/IX/V complex is a highly and specifically expressed VWF receptor on the platelet membrane with a crucial role in platelet adhesion at high shear rates. The cytosolic region of the GPIbα subunit interacts with the actin‐binding protein filamin A and with 14‐3‐3.^40^ Filamin A has emerged as a critical link between GPIbα and the actin cytoskeleton playing an important role in platelet stability and adhesion under high shear conditions.^41^ Filamin A also interacts with integrin αIIbβ3^42^ and has been involved in tyrosine kinase signaling during platelet activation possibly through anchoring of the tyrosine kinase Syk near the plasma membrane. PKA might impact indirectly on the GPIbα/filamin interaction by phosphorylating filamin A leading to its protection against proteolysis.^1^ A key site in GPIbα that is phosphorylated in resting platelets and dephosphorylated during activation by an unknown kinase and phosphatase, respectively, is S609.^43^ GPIbα S609 phosphorylation is required for 14‐3‐3 binding and receptor signaling.^44^ The GPIbβ subunit can be phosphorylated by PKA on S166 possibly contributing to binding of 14‐3‐3 to the GPIb/IX/V complex.^1^ S166 phosphorylation has been suggested to depend on localization of PKA to lipid rafts possibly via the A‐kinase anchoring protein moesin.^45^ The functional role of this phosphorylation is uncertain, as mutations of S166 have resulted in enhanced, as well as reduced receptor function,^1^ and the role of phosphorylated S166 for 14‐3‐3 binding has been questioned.^40^ PKA has been described to inhibit shedding of extracellular regions of GPIbα.^46^ Similarly, PKA inhibits shedding of the semaphorin family member Sema4D, a receptor that contributes to thrombus growth and stability.^47^ These shedding processes are mediated by the metalloproteinase ADAM17, however, the mechanisms involved in PKA‐induced inhibition of ADAM17 are not known. These data indicate a possible new role of PKA in the long‐term stabilization of certain membrane receptors which could contribute to thrombus growth.

### 2.4 GPVI

The GPVI‐Fc receptor γ chain complex plays a key role in platelet activation by collagen. Dimerization of the GPVI receptor is essential for binding of collagen to GPVI. GPVI activity depends on Gi signaling, indicating an inhibitory role of PKA. Indeed, cAMP and cGMP pathways inhibit GPVI dimerization.^48^ This suggests that inhibitory pathways might need to be down‐regulated in order to enable collagen binding to GPVI. Mathematical models of GPVI signaling have so far not taken this negative regulation by cyclic nucleotides into account.^49^

### 2.5 TxA_2_ receptor

The TxA_2_ receptor is a GPCR coupling to Gq and G13 and mediates platelet activation by TxA_2_. TxA_2_ is synthesized by cyclooxygenase‐1, the key target of the antiplatelet drug aspirin. The receptor can be phosphorylated by PKA and PKG leading to inhibited receptor signaling, although only limited evidence for the presence of this phosphorylation in platelets is available.^1^ An alternative mechanism of regulating TxA_2_‐induced Gq signaling involves the RGS18 complex described above. PKA‐induced phosphorylation of Gα13 linked to the TxA_2_ receptor might contribute to RhoA inhibition by cAMP.^1^

### 2.6 NO and sGC

The classical pathway for the activation of cGMP production involves endothelium‐derived NO stimulating platelet sGC. sGC represents the main receptor for endothelial NO in platelets and NO‐binding stimulates cGMP production up to 100‐fold.^50^ Interestingly, VWF can induce a 3‐fold increase in platelet cGMP levels,^51^ which has been attributed to an activation of sGC by Src kinase mediated Y192 phosphorylation^51^ and which might involve Akt kinase. Akt has also been suggested to play a role in lipopolysaccharide‐induced cGMP elevation.^52^ Thrombin and collagen might increase cGMP levels slightly involving Lyn kinase, although this has not been confirmed by other studies.^1^, ^53^ Platelet‐derived NO might contribute to sGC activation and platelet NO synthesis has recently been observed by microscopy.^54^ Stimulation of NO production by L‐nebivolol results in reduced thrombosis in mice,^55^ however, the presence of any NO‐synthase in platelets has been disputed,^51^, ^56^ indicating that NO might be released from other currently unknown sources. Cross‐activation of cGMP production by platelet activators like VWF has been interpreted in different ways, either as evidence of negative feedback or as an indication of a role for cGMP in platelet activation. A possible functional role of sGC during platelet activation is supported by studies of platelet specific sGC knockout mice and of patients lacking sGC expression. In both cases NO‐induced platelet inhibition is abolished, as expected.^57^, ^58^, ^59^ However, sGC knockout platelets aggregate less well in response to low agonist concentrations, they show reduced Akt and ERK activation, and sGC knockout mice exhibit prolonged bleeding and impaired thrombus formation.^57^ Similarly, platelets from patients lacking sGC expression do not aggregate well in response to low ADP concentrations, and platelet adhesion to collagen and VWF is attenuated.^59^ However, reduced sGC expression has also been linked to an increased risk of myocardial infarction possibly due to enhanced platelet activation.^60^ Interestingly, low NO concentrations have been seen to slightly potentiate thrombin‐induced Ca^2+^‐elevations thus potentially contributing to platelet activation.^61^, ^62^ Platelet activators like thrombospondin‐1 (TSP1) have been shown to cross‐inhibit sGC through some unknown mechanism.^63^ Further studies are required to clarify interactions between different receptors, NO, sGC, cGMP, and Ca^2+^ keeping in mind the methodological issues that might affect experiments on NO and cGMP pathways.^64^, ^65^

## 3 INTERACTIONS AT THE LEVEL OF SECOND MESSENGERS

### 3.1 Calcium

Gq‐coupled receptor and GPVI signaling trigger phospholipase C activation, leading to an increase in cytosolic inositol‐3‐phosphate (IP_3_) levels. IP_3_ binds to Ca^2+^ channels on the dense tubular system, called IP_3_‐receptors, resulting in Ca^2+^‐release into the cytosol followed by store‐operated calcium entry through the plasma membrane ultimately contributing to platelet activation.^66^ Steady state, ADP‐ and thrombin‐induced elevation of cytosolic Ca^2+^ levels have been modelled resulting in the validation of many static and kinetic parameters of the enzymes and ion channels involved.^67^, ^68^ The well established antagonism between activators and endothelial inhibitors in the regulation of Ca^2+^ levels has recently been confirmed in a systematic study comparing effects of ADP, thrombin, convulxin and U46619 in combination with activators of cAMP and cGMP pathways.^61^ The Gq GAP RGS18 contributes to differential control of Ca^2+^ levels by platelet activators and inhibitors.^7^, ^35^ PKA also inhibits IP_3_‐induced Ca^2+^‐release through phosphorylation of the IP_3_‐receptor and the IP_3_‐receptor‐associated G‐kinase substrate (IRAG, MRVI1).^1^ In the absence of IRAG cGMP is less potent in inhibiting platelet aggregation and dense granule release.^69^ The IP_3_‐receptor/PKG complex includes PDE type 5 which might support spatial control of cGMP signaling.^1^

### 3.2 Cyclic nucleotides

**Figure 6:**
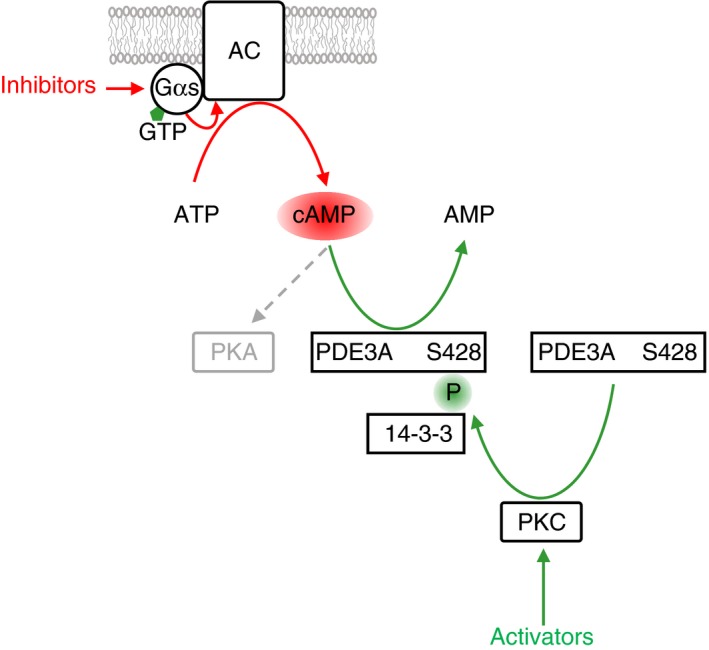
PDE3A signaling. Upon binding of active Gαs‐GTP adenylate cyclase (AC) is stimulated to synthesize cAMP from ATP. cAMP is required for the activation of cAMP‐dependent protein kinase (PKA). cAMP levels can be reduced by phosphodiesterase PDE3A which specifically degrades cAMP to AMP. PDE3A is regulated by multiple phosphorylation events. For example, platelet activation leads to activation of protein kinase C isoforms (PKC, Figure 4) which phosphorylate PDE3A on serine 428 leading to attachment of the phospho‐serine/threonine binding protein 14‐3‐3 and to stimulation of PDE3A function. In this way platelet activation interferes with inhibitory cAMP pathways

In addition to the cyclic nucleotide‐synthesizing enzymes AC and sGC, PDEs control the spatial and temporal distribution of cAMP and cGMP. Platelets contain three PDEs, two of which degrade cAMP (PDE2A and PDE3A) and one is cGMP‐specific (PDE5A).^1^ PDE3A inhibition by cilostazol or JF959602 leads to increased cAMP levels and inhibits the initial accumulation of platelets at sites of injury as well as attachment and detachment of platelets from growing thrombi in vivo.^70^ Thrombin stimulates the enzymatic function of PDE3A resulting in reduced cAMP levels.^11^ Thus, costimulation of Gq and Gi during platelet activation leads to PDE3A activation and AC inhibition resulting in a pronounced decrease in intracellular cAMP levels. Stimulation of PDE3A by thrombin is mediated by protein kinase C (PKC) which phosphorylates serines 312, 428, 438, 465, and 492 of PDE3A.^71^ A similar phosphorylation pattern is induced by treatment of platelets with collagen‐related peptide or by U46619. Phosphorylated S428 has been suggested to play a role as gate‐keeper for binding of 14‐3‐3 to PDE3A (Figure 6). In contrast, the cAMP/PKA pathway triggers the phosphorylation of S312 alone which is not linked to 14‐3‐3 binding but which also stimulates the enzymatic function of PDE3A. These data indicate that activating pathways rapidly phosphorylate and stimulate PDE3A to reduce cAMP levels below an inhibitory threshold whereas inhibitory PKA pathways may use PDE3A in a negative feedback loop. 14‐3‐3 binding to PDE3A might help to discriminate between activating and inhibitory pathways.^71^ Modelling of cyclic nucleotide levels confirms that PDEs are highly regulated exhibiting very low basal catalytic activity.^72^ These studies indicate that cAMP does not influence total cGMP levels. In contrast, cGMP might stimulate PKA function through an inhibition of PDE3A.^73^ In fact, a synergistic inhibitory role for NO/cGMP in the cAMP/PKA pathway has been proposed.^74^, ^75^ Subcellular compartmentalization of cyclic nucleotide signaling by scaffolding proteins like kinase anchoring proteins is likely to have an important role in determining crosstalk between NO/cGMP and cAMP pathways.^1^

## 4 INTERACTIONS INVOLVING KINASES AND PHOSPHATASES

### 4.1 Kinases

Opposite regulation of MLC kinase and MLC phosphorylation levels by activating and cyclic nucleotide‐mediated inhibitory pathways has been known for a long time,^76^ and this regulation is likely to contribute to the control of actin dynamics, platelet adhesion, and aggregation.^77^ Extracellular signal‐regulated kinase (ERK) and p38 mitogen‐activated protein kinase (MAPK) activation induced by VWF, thrombin, collagen and TxA_2_ are generally thought to be blocked by cyclic nucleotide pathways,^78^ although PKG‐mediated activation of p38 MAPK has also been described.^1^ Thrombin and collagen are able to activate PKA by releasing the catalytic PKA subunit from an NFκB complex possibly involving PI3K resulting in negative feedback and dampened platelet activation.^79^ Recently, PKA activation was detected during thrombus formation. Using biosensors based on the principle of Förster resonance energy transfer expressed in mouse platelets, Hiratsuka et al 80 observed ERK activation throughout the developing thrombus after laser injury of subcutaneous arterioles, as expected. Surprisingly, PKA activation was detectable inside the thrombus as well, both in the vasculature in vivo and in a flow chamber in the absence of endothelium. PKA appeared to be more active on the downstream side of the aggregate, possibly matching the outer shell structure where P2Y12 signaling has been suggested to be involved in regulating thrombus size.^27^ Thus, PKA might be activated during thrombus formation by an endothelium‐independent mechanism to limit thrombus growth.

Platelet activation by oxidized low‐density lipoproteins has been suggested to involve Src kinases, PKC, p47^phox^ phosphorylation, NADPH oxidase 2, and the production of reactive oxygen species ultimately leading to an inhibition of PKG.^81^ This mechanism might be similar to TSP1‐induced inhibition of PKA which has also been suggested to involve Src family kinases.^82^ Src family kinases are crucial for initiating and propagating signals from major platelet recepors,^83^ supporting the concept that platelet activation requires blockage of cyclic nucleotide signaling. Recent mass spectrometry based proteome studies indicate that many kinases are phosphorylated upon PGI_2_ and/or ADP treatment of platelets probably leading to complex inhibition/activation patterns.^18^, ^19^, ^84^

### 4.2 Phosphatases

Proteome studies document de‐phosphorylation of many sites confirming an important role for phosphatases in platelet control.^18^, ^85^ Certain kinases are able to activate phosphatases, as shown for thrombin‐induced phosphorylation and activation of SHP‐1 leading to de‐phosphorylation of spinophilin,^35^ and for PKA‐mediated activation of PP1 leading to detachment of 14‐3‐3 from RGS18.^38^ Although tyrosine phosphatases are important during platelet activation,^86^ their role in cyclic nucleotide‐mediated platelet inhibition remains unclear. Interestingly, of the many phosphorylation sites induced by ADP treatment only half are reversed upon activation of cAMP pathways while the remaining sites affected by ADP are not altered. However, these cAMP‐induced changes are sufficient to reverse ADP‐induced platelet aggregation.^18^

## 5 INTERACTIONS INVOLVING SMALL G‐PROTEINS

### 5.1 Rap1

**Figure 7:**
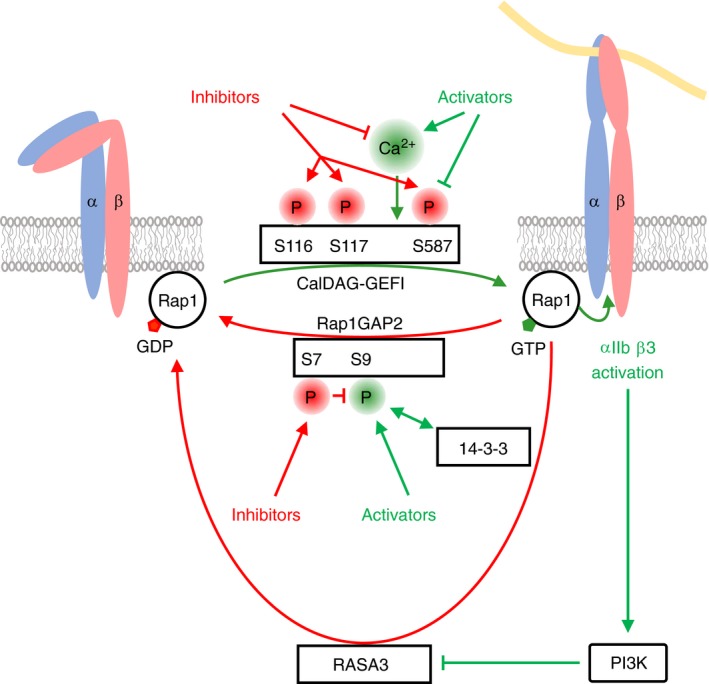
Regulation of Rap1 by GEFs and GAPs. Integrin αIIbß3 is a major platelet integrin required for platelet aggregation. The small G‐protein Rap1 is a positive regulator of integrin αIIbß3 activation and cycles between an inactive, GDP‐bound and an active, GTP‐bound state. Formation of Rap1‐GTP and integrin activation are enabled by the calcium ion (Ca^2+^) dependent guanine‐nucleotide exchange factor CalDAG‐GEFI, whereas hydrolysis of Rap1‐GTP to inactive Rap1‐GDP requires the GTPase‐activating proteins (GAPs) Rap1GAP2 and RASA3. Rap1GAP2 interacts with the phospho‐serine/threonine binding protein 14‐3‐3 through phosphorylated serine 9 on Rap1GAP2. During platelet activation levels of free intracellular Ca^2+^ rise (Figure 4) leading to the activation of CalDAG‐GEFI, increased Rap1‐GTP formation and integrin activation. In contrast, cyclic nucleotide‐dependent inhibitory pathways on one hand suppress intracellular Ca^2+^ levels, thus indirectly inhibit CalDAG‐GEFI activation, and on the other hand result in phosphorylation of CalDAG‐GEFI on serines 116, 117 and 587 inhibiting its activity. Simultaneously, cyclic nucleotide‐dependent inhibitory pathways induce Rap1GAP2 phosphorylation on serine 7 leading to an inhibition of 14‐3‐3 binding, activation of Rap1GAP2, reduced Rap1‐GTP levels and lowered Rap1 activity. In contrast, platelet activation leads to phosphorylation of Rap1GAP2 on serine 9 resulting in enhanced 14‐3‐3 binding and inhibition of Rap1GAP2. In parallel, αIIbβ3‐mediated outside‐in signaling leads to phosphatidylinositol 3‐kinase (PI3K) activation, which in turn reduces the GAP activity of RASA3. Platelet activators also suppress the phosphorylation of the inhibitory serine 587 site on CalDAG‐GEFI. The consequence of Rap1GAP2 and RASA3 inhibition, along with CalDAG‐GEFI stimulation is enhanced Rap1 activity. Phosphorylation sites linked to platelet inhibition are highlighted in red, whereas sites linked to platelet activation are marked in green

Small G‐proteins of the Ras and Rho families like Rap1, Rac1, and RhoA are critical regulators of platelet function.^87^ Similarly to heterotrimeric G‐proteins, small G‐proteins are dependent on specific GEFs and GAPs for activation and inhibition, respectively.^88^, ^89^ These GEFs and GAPs have been shown to be controlled by activation pathways as well as by cyclic nucleotide signaling leading to differential changes in small G‐protein activity. Rap1 is the most highly expressed small G protein in platelets and is the main activator of the fibrinogen receptor integrin αIIbβ3. CalDAG‐GEFI (CD‐GEFI, RasGRP2), an important GEF of Rap1, is activated by Gq‐coupled receptor signaling leading to increased Rap1‐GTP levels and integrin αIIbβ3 activation (Figure 7). PKA inhibits CalDAG‐GEFI by phosphorylation of S116, S117, and S587 resulting in lowered Rap1‐GTP.^90^, ^91^ ADP treatment is also able to reduce S587 phosphorylation thus reversing PKA effects.^18^ A corresponding regulation of Rap1GAP2, a GAP of Rap1, contributes to the control of Rap1 activity. Rap1GAP2 function is blocked by platelet activators and activated by cyclic nucleotide pathways which involves 14‐3‐3 binding. During platelet activation Rap1GAP2 S9 phosphorylation enhances 14‐3‐3 binding, whereas PKA/PKG‐mediated S7 phosphorylation does the opposite (Figure 7).^92^ Platelet activation has also been shown to inhibit the function of RASA3, a highly expressed GAP of Rap1 in platelets, in a PI3K‐dependent manner.^21^, ^22^ Vasodilator‐stimulated phosphoprotein (VASP) is an actin filament and focal adhesion binding protein and was one of the first substrates of PKA/PKG to be identified in platelets. PKA and PKG phosphorylate VASP preferentially on serines 157 and 239, respectively.^1^ Recent data suggest that VASP plays a positive role in the activation of the small G‐protein Rap1. Treatment of VASP‐deficient mouse platelets with thrombin, ADP or U46619 results in reduced Rap1‐GTP levels compared to normal platelets.^93^ VASP‐dependent Rap1 activation might be mediated through an interaction of VASP with Crk‐like, an activator of C3G (RapGEF1), which is a GEF of Rap1. PKA‐dependent VASP S157 phosphorylation inhibits the VASP/Crkl interaction thus potentially contributing to platelet inhibition.^93^

### 5.2 Rac1

Rac1 integrates signals from several platelet activation pathways and is involved in platelet lamellipodia formation and thrombus stability. Similar to Rap1, parallel regulation of the Rac1 regulating proteins ArhGEF6 and ArhGAP17 has been described whereby PKA and PKG simultaneously inhibit ArhGEF6 and stimulate ArhGAP17 leading to attenuated Rac1 function.^94^ These effects are mediated by ArhGEF6 S684 phosphorylation and 14‐3‐3 binding, and by ArhGAP17 S702 phosphorylation resulting in detachment of the actin regulator Cdc42‐interacting protein 4, CIP4.

### 5.3 RhoA

RhoA is activated downstream of G13 and is an essential regulator of platelet shape change. RhoA is also known to be differentially regulated by cyclic nucleotides and platelet activators.^95^ RhoA inhibition by PKA/PKG involves RhoA S188 phosphorylation and reduced association of RhoA with ROCK2 and the myosin‐binding regulatory subunit of PP1, MYPT1.^77^, ^96^

### 5.4 Arf6

ADP‐ribosylation factor 6 (Arf6) belongs to the Arf family of small G‐proteins and is involved in receptor trafficking in platelets.^97^ In contrast to Rap1, Rac1, and RhoA, Arf6 is inhibited during platelet activation, whereas cyclic nucleotide pathways elevate Arf6‐GTP levels.^98^ Cytohesin‐2 is a GEF for Arf6 which plays a role in the constitutive inhibition of dense granule secrection in resting platelets.^99^ Cytohesin‐2 is phosphorylated by PKC during platelet activation leading to the dissociation of Arf6 and its GEF^99^ and might be involved in the opposing regulation of Arf6‐GTP levels during platelet activation and inhibition. Phosphoproteome studies indicate that many other GEFs and GAPs might play important roles in PGI_2_‐ and ADP‐mediated platelet regulation.^18^

## 6 CLINICAL IMPLICATIONS

Inherited disorders affecting genes involved in the positive or negative regulation of platelet cyclic nucleotide levels underscore the importance of this regulatory system to hemostasis and thrombosis in humans. For example, Gs hyperfunction leads to an enhanced tendency for bleeding,^100^ enhanced NO signaling correlates with reduced risk of thrombosis,^101^ whereas Gs or sGC dysfunction culminates in enhanced susceptibility to thrombosis.^60^, ^102^ In particular, loss of sGC expression, through a combination of mutations in the α1 subunit of sGC and in the sGC‐interacting chaperone CCTη, was shown to reduce platelet cGMP levels and to increase the risk of myocardial infarction.^60^ Recently, a meta‐analysis identified PDE3A as a novel stroke risk locus.^103^

The clinical relevance of crosstalk between activating and cyclic nucleotide‐dependent inhibitory pathways is further highlighted by the success of drugs targeting either the Gi‐coupled P2Y12 receptor or PDEs 3A and 5A,^12^, ^104^, ^105^ which prevent the blockade of cyclic nucleotide production or degradation, respectively.^106^ Stimulators of the cGMP pathway are commonly used to treat vascular disease due to their ability to lower vascular tone by acting on smooth muscle cells, however, simultaneous platelet inhibition might contribute to their beneficial effects. For example, NO donors are employed in ischaemic heart disease,^107^ and the sGC stimulator riociguat has recently been developed as a new treatment for thromboembolic pulmonary hypertension.^108^ Besides acting as a vasodilator, riociguat promotes platelet inhbition by acting synergistically with PGI_2_.^75^ The state of inhibitory pathways can be monitored by measuring intracellular phosphorylation of the PKA/PKG substrate protein VASP in platelets, which has been used to study the efficiency of P2Y12 inhibition in patients.^109^, ^110^ Pharmacological studies of P2Y12 receptor based anti‐platelet therapy show that addition of low concentrations of PGI_2_ and NO to platelets from volunteers receiving dual anti‐platelet therapy with aspirin and the P2Y12 blocker prasugrel strongly enhances platelet inhibition in the presence of TRAP‐6 or collagen.^8^ This synergy between P2Y12 inhibitors and cAMP/cGMP signaling suggests that in the presence of similar levels of platelet blockade thrombosis risk might critically depend on the availability of PGI_2_ and NO and thus on endothelial function. Since endothelial dysfunction is commonly observed in patients with cardiovascular disease it could be beneficial to include endothelial function testing in the assessment of anti‐platelet therapy.^8^ Combining stimulators of endothelium‐dependent cAMP pathways such as the PDE3A inhibitor cilostazol with P2Y12 inhibitors might improve anti‐thrombotic protection.^17^ Interestingly, cilostazol treatment has been associated with fewer hemorrhagic events compared to aspirin.^104^, ^111^ Although P2Y12 and PDE inhibitors are widely used in anti‐platelet therapy, their downstream effects on the platelet signaling network are unappreciated, and likely involve systems‐level changes, including altered phosphorylation of many PKA substrate proteins and their functional implications.

## 7 CONCLUSIONS

Cyclic nucleotide‐mediated inhibitory signaling is emerging to be closely intertwined with activating pathways. On the other hand, effective platelet activation mandates interference with inhibitory pathways at multiple levels, including blocking cyclic nucleotide production and promoting their degradation, and dephosphorylating PKA substrates. Inhibitors and activators tend to regulate the same signaling nodes in opposite directions (Table 1). Larger protein assemblies such as the RGS18/spinophilin/14‐3‐3/SHP‐1/PP1 or GPIb/IX/V/14‐3‐3/filaminA/actin/Syk complexes have been revealed which are likely to facilitate spatial and temporal coordination of signaling. Many of the described signaling processes reorganize protein‐protein interactions by multiple phosphorylation and de‐phosphorylation events often involving binding or detachment of 14‐3‐3 proteins. GEFs and GAPs are becoming apparent as major targets in the control of small G‐proteins. Focused studies of individual proteins, phosphorylation sites and kinases in cells and animal models as well as proteomics approaches combined with computational analyses will be required to gain deeper insights into patterns of platelet regulation. Although knockout mouse studies are essential in our understanding of these patterns, to accurately interpret results it is important to consider possible compensatory changes in gene expression. This issue is particulary relevant when analyzing interactions between positive and negative signals, and it is prudent to verify if the expression of related proteins involved in the same or the opposite pathway are altered which could contribute to the phenotype.^112^ New information on the topology of platelet signaling networks and the crossover points between cyclic nucleotide inhibition and platelet activation will likely result in either new drug targets or better combinations of existing drugs.

## RELATIONSHIP DISCLOSURE

Authors have nothing to disclose.

## AUTHOR CONTRIBUTIONS

Z.N. and A.S. wrote the paper.

## References

[1] Smolenski A . Novel roles of cAMP/cGMP‐dependent signaling in platelets. J Thromb Haemost. 2012;10:167–76. 22136590 10.1111/j.1538-7836.2011.04576.x

[2] Walter U , Gambaryan S . cGMP and cGMP‐dependent protein kinase in platelets and blood cells. Handb Exp Pharmacol. 2009;191:533–48. 10.1007/978-3-540-68964-5_23 19089344

[3] Clemetson KJ . Platelets and primary haemostasis. Thromb Res. 2012;129:220–4. 22178577 10.1016/j.thromres.2011.11.036

[4] Brass LF , Tomaiuolo M , Stalker TJ . Harnessing the platelet signaling network to produce an optimal hemostatic response. Hematol Oncol Clin North Am. 2013;27:381–409. 23714305 10.1016/j.hoc.2013.02.002 PMC3787971

[5] Bye AP , Unsworth AJ , Gibbins JM . Platelet signaling: a complex interplay between inhibitory and activatory networks. J Thromb Haemost. 2016;14:918–30. 26929147 10.1111/jth.13302 PMC4879507

[6] Coxon CH , Geer MJ , Senis YA . ITIM receptors: more than just inhibitors of platelet activation. Blood. 2017;129:3407–18. 28465343 10.1182/blood-2016-12-720185 PMC5562394

[7] Gegenbauer K , Elia G , Blanco‐Fernandez A , Smolenski A . Regulator of G‐protein signaling 18 integrates activating and inhibitory signaling in platelets. Blood. 2012;119:3799–807. 22234696 10.1182/blood-2011-11-390369

[8] Chan MV , Knowles RB , Lundberg MH , et al. P2Y12 receptor blockade synergizes strongly with nitric oxide and prostacyclin to inhibit platelet activation. Br J Clin Pharmacol. 2016;81:621–33. 26561399 10.1111/bcp.12826 PMC4799935

[9] Kirkby NS , Lundberg MH , Chan MV , et al. Blockade of the purinergic P2Y12 receptor greatly increases the platelet inhibitory actions of nitric oxide. Proc Natl Acad Sci USA. 2013;110:15782–7. 24003163 10.1073/pnas.1218880110 PMC3785756

[10] Signarvic RS , Cierniewska A , Stalker TJ , et al. RGS/Gi2alpha interactions modulate platelet accumulation and thrombus formation at sites of vascular injury. Blood. 2010;116:6092–100. 20852125 10.1182/blood-2010-05-283846 PMC3031394

[11] Zhang W , Colman RW . Thrombin regulates intracellular cyclic AMP concentration in human platelets through phosphorylation/activation of phosphodiesterase 3A. Blood. 2007;110:1475–82. 17392505 10.1182/blood-2006-10-052522 PMC1975837

[12] Michelson AD , Bhatt DL . How I use laboratory monitoring of antiplatelet therapy. Blood. 2017;130:713–21. 28600334 10.1182/blood-2017-03-742338

[13] Rao GH , Reddy KR , White JG . The influence of epinephrine on prostacyclin (PGI2) induced dissociation of ADP aggregated plateletes. Prostaglandins Med. 1980;4:385–97. 6251492 10.1016/0161-4630(80)90047-6

[14] Stamler JS , Vaughan DE , Loscalzo J . Synergistic disaggregation of platelets by tissue‐type plasminogen activator, prostaglandin E1, and nitroglycerin. Circ Res. 1989;65:796–804. 2504509 10.1161/01.res.65.3.796

[15] White JG , Rao GH . Aggregated‐disaggregated, refractory platelets retain sensitivity to ristocetin. Thromb Res. 1996;84:253–66. 8948050 10.1016/s0049-3848(96)00185-5

[16] Kikura M , Kazama T , Ikeda T , Sato S . Disaggregatory effects of prostaglandin E1, amrinone and milrinone on platelet aggregation in human whole blood. Platelets. 2000;11:446–58. 11177444 10.1080/09537100020027824

[17] Warner TD , Armstrong PC , Chan MV , Knowles RB . The importance of endothelium‐derived mediators to the efficacy of dual anti‐platelet therapy. Expert Rev Hematol. 2016;9:223–5. 26822235 10.1586/17474086.2016.1140035

[18] Beck F , Geiger J , Gambaryan S , et al. Temporal quantitative phosphoproteomics of ADP stimulation reveals novel central nodes in platelet activation and inhibition. Blood. 2017;129:e1–12. 28060719 10.1182/blood-2016-05-714048 PMC5248936

[19] Beck F , Geiger J , Gambaryan S , et al. Time‐resolved characterization of cAMP/PKA‐dependent signaling reveals that platelet inhibition is a concerted process involving multiple signaling pathways. Blood. 2014;123:e1–10. 24324209 10.1182/blood-2013-07-512384

[20] Guidetti GF , Canobbio I , Torti M . PI3K/Akt in platelet integrin signaling and implications in thrombosis. Adv Biol Regul. 2015;59:36–52. 26159296 10.1016/j.jbior.2015.06.001

[21] Stefanini L , Paul DS , Robledo RF , et al. RASA3 is a critical inhibitor of RAP1‐dependent platelet activation. J Clin Invest. 2015;125:1419–32. 25705885 10.1172/JCI77993 PMC4396462

[22] Battram AM , Durrant TN , Agbani EO , et al. The phosphatidylinositol 3,4,5‐trisphosphate (PI(3,4,5)P3) binder Rasa3 regulates phosphoinositide 3‐kinase (PI3K)‐dependent integrin alphaIIbbeta3 outside‐in signaling. J Biol Chem. 2017;292:1691–704. 27903653 10.1074/jbc.M116.746867 PMC5290945

[23] Cattaneo M , Lecchi A . Inhibition of the platelet P2Y12 receptor for adenosine diphosphate potentiates the antiplatelet effect of prostacyclin. J Thromb Haemost. 2007;5:577–82. 17155953 10.1111/j.1538-7836.2007.02356.x

[24] Foster CJ , Prosser DM , Agans JM , et al. Molecular identification and characterization of the platelet ADP receptor targeted by thienopyridine antithrombotic drugs. J Clin Invest. 2001;107:1591–8. 11413167 10.1172/JCI12242 PMC200194

[25] Hollopeter G , Jantzen HM , Vincent D , et al. Identification of the platelet ADP receptor targeted by antithrombotic drugs. Nature. 2001;409:202–7. 11196645 10.1038/35051599

[26] Welsh JD , Muthard RW , Stalker TJ , Taliaferro JP , Diamond SL , Brass LF . A systems approach to hemostasis: 4. How hemostatic thrombi limit the loss of plasma‐borne molecules from the microvasculature. Blood. 2016;127:1598–605. 26738537 10.1182/blood-2015-09-672188 PMC4807424

[27] Stalker TJ , Traxler EA , Wu J , et al. Hierarchical organization in the hemostatic response and its relationship to the platelet‐signaling network. Blood. 2013;121:1875–85. 23303817 10.1182/blood-2012-09-457739 PMC3591806

[28] Hubertus K , Mischnik M , Timmer J , et al. Reciprocal regulation of human platelet function by endogenous prostanoids and through multiple prostanoid receptors. Eur J Pharmacol. 2014;740:15–27. 25003953 10.1016/j.ejphar.2014.06.030

[29] Mischnik M , Hubertus K , Geiger J , Dandekar T , Timmer J . Dynamical modelling of prostaglandin signalling in platelets reveals individual receptor contributions and feedback properties. Mol BioSyst. 2013;9:2520–9. 23903629 10.1039/c3mb70142e

[30] Kowalska MA , Ratajczak MZ , Majka M , et al. Stromal cell‐derived factor‐1 and macrophage‐derived chemokine: 2 chemokines that activate platelets. Blood. 2000;96:50–7. 10891429

[31] Walsh TG , Harper MT , Poole AW . SDF‐1alpha is a novel autocrine activator of platelets operating through its receptor CXCR4. Cell Signal. 2015;27:37–46. 25283599 10.1016/j.cellsig.2014.09.021 PMC4265729

[32] Sebastiano M , Momi S , Falcinelli E , Bury L , Hoylaerts MF , Gresele P . A novel mechanism regulating human platelet activation by MMP‐2‐mediated PAR1 biased signaling. Blood. 2017;129:883–95. 28034890 10.1182/blood-2016-06-724245

[33] Delesque‐Touchard N , Pendaries C , Volle‐Challier C , et al. Regulator of G‐protein signaling 18 controls both platelet generation and function. PLoS One. 2014;9:e113215. 25405900 10.1371/journal.pone.0113215 PMC4236145

[34] Alshbool FZ , Karim ZA , Vemana HP , Conlon C , Lin OA , Khasawneh FT . The regulator of G‐protein signaling 18 regulates platelet aggregation, hemostasis and thrombosis. Biochem Biophys Res Commun. 2015;462:378–82. 25969426 10.1016/j.bbrc.2015.04.143

[35] Ma P , Cierniewska A , Signarvic R , et al. A newly identified complex of spinophilin and the tyrosine phosphatase, SHP‐1, modulates platelet activation by regulating G protein‐dependent signaling. Blood. 2012;119:1935–45. 22210881 10.1182/blood-2011-10-387910 PMC3293648

[36] Ma P , Foote DC , Sinnamon AJ , Brass LF . Dissociation of SHP‐1 from spinophilin during platelet activation exposes an inhibitory binding site for protein phosphatase‐1 (PP1). PLoS One. 2015;10:e0119496. 25785436 10.1371/journal.pone.0119496 PMC4364895

[37] Ma P , Ou K , Sinnamon AJ , Jiang H , Siderovski DP , Brass LF . Modulating platelet reactivity through control of RGS18 availability. Blood. 2015;126:2611–20. 26407691 10.1182/blood-2015-04-640037 PMC4671108

[38] Gegenbauer K , Nagy Z , Smolenski A . Cyclic nucleotide dependent dephosphorylation of regulator of G‐protein signaling 18 in human platelets. PLoS One. 2013;8:e80251. 24244663 10.1371/journal.pone.0080251 PMC3820651

[39] Mazharian A , Mori J , Wang YJ , et al. Megakaryocyte‐specific deletion of the protein‐tyrosine phosphatases Shp1 and Shp2 causes abnormal megakaryocyte development, platelet production, and function. Blood. 2013;121:4205–20. 23509158 10.1182/blood-2012-08-449272 PMC3656453

[40] Mangin PH , Receveur N , Wurtz V , David T , Gachet C , Lanza F . Identification of five novel 14‐3‐3 isoforms interacting with the GPIb‐IX complex in platelets. J Thromb Haemost. 2009;7:1550–5. 19558434 10.1111/j.1538-7836.2009.03530.x

[41] Jurak Begonja A , Hoffmeister KM , Hartwig JH , Falet H . FlnA‐null megakaryocytes prematurely release large and fragile platelets that circulate poorly. Blood. 2011;118:2285–95. 21652675 10.1182/blood-2011-04-348482 PMC3162356

[42] Falet H . New insights into the versatile roles of platelet FlnA. Platelets. 2013;24:1–5. 22372530 10.3109/09537104.2011.654004 PMC6781629

[43] Mangin P , David T , Lavaud V , et al. Identification of a novel 14‐3‐3zeta binding site within the cytoplasmic tail of platelet glycoprotein Ibalpha. Blood. 2004;104:420–7. 15054037 10.1182/blood-2003-08-2881

[44] Estevez B , Kim K , Delaney MK , et al. Signaling‐mediated cooperativity between glycoprotein Ib‐IX and protease‐activated receptors in thrombin‐induced platelet activation. Blood. 2016;127:626–36. 26585954 10.1182/blood-2015-04-638387 PMC4742550

[45] Raslan Z , Magwenzi S , Aburima A , Tasken K , Naseem KM . Targeting of type I protein kinase A to lipid rafts is required for platelet inhibition by the 3′,5′‐cyclic adenosine monophosphate‐signaling pathway. J Thromb Haemost. 2015;13:1721–34. 26176741 10.1111/jth.13042

[46] Dai K , Yan R , Li S , Fan Y , Zhuang F , Ruan C . Prolonged inhibition of protein kinase A results in metalloproteinase‐dependent platelet GPIbalpha shedding. Thromb Res. 2009;124:101–9. 19181367 10.1016/j.thromres.2008.12.044

[47] Chen T , Xu DZ , Li Q , et al. The regulation of Sema4D exodomain shedding by protein kinase A in platelets. Platelets. 2016;27:673–9. 27809714 10.3109/09537104.2016.1154141

[48] Loyau S , Dumont B , Ollivier V , et al. Platelet glycoprotein VI dimerization, an active process inducing receptor competence, is an indicator of platelet reactivity. Arterioscler Thromb Vasc Biol. 2012;32:778–85. 22155453 10.1161/ATVBAHA.111.241067

[49] Dunster JL , Mazet F , Fry MJ , Gibbins JM , Tindall MJ . Regulation of early steps of GPVI signal transduction by phosphatases: a systems biology approach. PLoS Comput Biol. 2015;11:e1004589. 26584182 10.1371/journal.pcbi.1004589 PMC4652868

[50] Mullershausen F , Russwurm M , Thompson WJ , Liu L , Koesling D , Friebe A . Rapid nitric oxide‐induced desensitization of the cGMP response is caused by increased activity of phosphodiesterase type 5 paralleled by phosphorylation of the enzyme. J Cell Biol. 2001;155:271–8. 11604422 10.1083/jcb.200107001 PMC2198829

[51] Gambaryan S , Kobsar A , Hartmann S , et al. NO‐synthase‐/NO‐independent regulation of human and murine platelet soluble guanylyl cyclase activity. J Thromb Haemost. 2008;6:1376–84. 18485089 10.1111/j.1538-7836.2008.03014.x

[52] Lopes‐Pires ME , Naime AC , Almeida Cardelli NJ , Anjos DJ , Antunes E , Marcondes S . PKC and AKT modulate cGMP/PKG signaling pathway on platelet aggregation in experimental sepsis. PLoS One. 2015;10:e0137901. 26375024 10.1371/journal.pone.0137901 PMC4573322

[53] Nygaard G , Herfindal L , Kopperud R , et al. Time‐dependent inhibitory effects of cGMP‐analogues on thrombin‐induced platelet‐derived microparticles formation, platelet aggregation, and P‐selectin expression. Biochem Biophys Res Commun. 2014;449:357–63. 24845383 10.1016/j.bbrc.2014.05.032

[54] Cozzi MR , Guglielmini G , Battiston M , et al. Visualization of nitric oxide production by individual platelets during adhesion in flowing blood. Blood. 2015;125:697–705. 25480660 10.1182/blood-2014-06-579474

[55] Momi S , Caracchini R , Falcinelli E , Evangelista S , Gresele P . Stimulation of platelet nitric oxide production by nebivolol prevents thrombosis. Arterioscler Thromb Vasc Biol. 2014;34:820–9. 24558107 10.1161/ATVBAHA.114.303290

[56] Ozuyaman B , Godecke A , Kusters S , Kirchhoff E , Scharf RE , Schrader J . Endothelial nitric oxide synthase plays a minor role in inhibition of arterial thrombus formation. Thromb Haemost. 2005;93:1161–7. 15968403 10.1160/TH03-09-0588

[57] Zhang G , Xiang B , Dong A , et al. Biphasic roles for soluble guanylyl cyclase (sGC) in platelet activation. Blood. 2011;118:3670–9. 21803853 10.1182/blood-2011-03-341107 PMC3186338

[58] Rukoyatkina N , Walter U , Friebe A , Gambaryan S . Differentiation of cGMP‐dependent and ‐independent nitric oxide effects on platelet apoptosis and reactive oxygen species production using platelets lacking soluble guanylyl cyclase. Thromb Haemost. 2011;106:922–33. 21800013 10.1160/TH11-05-0319

[59] Herve D , Philippi A , Belbouab R , et al. Loss of alpha1beta1 soluble guanylate cyclase, the major nitric oxide receptor, leads to moyamoya and achalasia. Am J Hum Genet. 2014;94:385–94. 24581742 10.1016/j.ajhg.2014.01.018 PMC3951937

[60] Erdmann J , Stark K , Esslinger UB , et al. Dysfunctional nitric oxide signalling increases risk of myocardial infarction. Nature. 2013;504:432–6. 24213632 10.1038/nature12722

[61] Lee MY , Diamond SL . A human platelet calcium calculator trained by pairwise agonist scanning. PLoS Comput Biol. 2015;11:e1004118. 25723389 10.1371/journal.pcbi.1004118 PMC4344206

[62] Blackmore PF . Biphasic effects of nitric oxide on calcium influx in human platelets. Thromb Res. 2011;127:e8–14. 21056902 10.1016/j.thromres.2010.10.002

[63] Isenberg JS , Romeo MJ , Yu C , et al. Thrombospondin‐1 stimulates platelet aggregation by blocking the antithrombotic activity of nitric oxide/cGMP signaling. Blood. 2008;111:613–23. 17890448 10.1182/blood-2007-06-098392 PMC2200855

[64] Gambaryan S , Tsikas D . A review and discussion of platelet nitric oxide and nitric oxide synthase: do blood platelets produce nitric oxide from L‐arginine or nitrite? Amino Acids. 2015;47:1779–93. 25929585 10.1007/s00726-015-1986-1

[65] Russo I , Del Mese P , Viretto M , et al. Sodium azide, a bacteriostatic preservative contained in commercially available laboratory reagents, influences the responses of human platelets via the cGMP/PKG/VASP pathway. Clin Biochem. 2008;41:343–9. 18022387 10.1016/j.clinbiochem.2007.10.012

[66] Varga‐Szabo D , Braun A , Nieswandt B . Calcium signaling in platelets. J Thromb Haemost. 2009;7:1057–66. 19422456 10.1111/j.1538-7836.2009.03455.x

[67] Dolan AT , Diamond SL . Systems modeling of Ca(2+) homeostasis and mobilization in platelets mediated by IP3 and store‐operated Ca(2+) entry. Biophys J. 2014;106:2049–60. 24806937 10.1016/j.bpj.2014.03.028 PMC4017292

[68] Sveshnikova AN , Balatskiy AV , Demianova AS , et al. Systems biology insights into the meaning of the platelet's dual‐receptor thrombin signaling. J Thromb Haemost. 2016;14:2045–57. 27513817 10.1111/jth.13442

[69] Schinner E , Salb K , Schlossmann J . Signaling via IRAG is essential for NO/cGMP‐dependent inhibition of platelet activation. Platelets. 2011;22:217–27. 21244222 10.3109/09537104.2010.544151

[70] Sim DS , Merrill‐Skoloff G , Furie BC , Furie B , Flaumenhaft R . Initial accumulation of platelets during arterial thrombus formation in vivo is inhibited by elevation of basal cAMP levels. Blood. 2004;103:2127–34. 14645013 10.1182/blood-2003-04-1133

[71] Hunter RW , Mackintosh C , Hers I . Protein kinase C‐mediated phosphorylation and activation of PDE3A regulate cAMP levels in human platelets. J Biol Chem. 2009;284:12339–48. 19261611 10.1074/jbc.M807536200 PMC2673302

[72] Wangorsch G , Butt E , Mark R , et al. Time‐resolved in silico modeling of fine‐tuned cAMP signaling in platelets: feedback loops, titrated phosphorylations and pharmacological modulation. BMC Syst Biol. 2011;5:178. 22034949 10.1186/1752-0509-5-178 PMC3247139

[73] Dunkern TR , Hatzelmann A . The effect of Sildenafil on human platelet secretory function is controlled by a complex interplay between phosphodiesterases 2, 3 and 5. Cell Signal. 2005;17:331–9. 15567064 10.1016/j.cellsig.2004.07.007

[74] Jensen BO , Kleppe R , Kopperud R , et al. Dipyridamole synergizes with nitric oxide to prolong inhibition of thrombin‐induced platelet shape change. Platelets. 2011;22:8–19. 20958117 10.3109/09537104.2010.517581

[75] Reiss C , Mindukshev I , Bischoff V , et al. The sGC stimulator riociguat inhibits platelet function in washed platelets but not in whole blood. Br J Pharmacol. 2015;172:5199–210. 26282717 10.1111/bph.13286 PMC4687809

[76] Nishikawa M , de Lanerolle P , Lincoln TM , Adelstein RS . Phosphorylation of mammalian myosin light chain kinases by the catalytic subunit of cyclic AMP‐dependent protein kinase and by cyclic GMP‐dependent protein kinase. J Biol Chem. 1984;259:8429–36. 6547441

[77] Aburima A , Wraith KS , Raslan Z , Law R , Magwenzi S , Naseem KM . cAMP signaling regulates platelet myosin light chain (MLC) phosphorylation and shape change through targeting the RhoA‐Rho kinase‐MLC phosphatase signaling pathway. Blood. 2013;122:3533–45. 24100445 10.1182/blood-2013-03-487850

[78] Schwarz UR , Kobsar AL , Koksch M , Walter U , Eigenthaler M . Inhibition of agonist‐induced p42 and p38 mitogen‐activated protein kinase phosphorylation and CD40 ligand/P‐selectin expression by cyclic nucleotide‐regulated pathways in human platelets. Biochem Pharmacol. 2000;60:1399–407. 11008134 10.1016/s0006-2952(00)00452-4

[79] Gambaryan S , Kobsar A , Rukoyatkina N , et al. Thrombin and collagen induce a feedback inhibitory signaling pathway in platelets involving dissociation of the catalytic subunit of protein kinase A from an NFkappaB‐IkappaB complex. J Biol Chem. 2010;285:18352–63. 20356841 10.1074/jbc.M109.077602 PMC2881761

[80] Hiratsuka T , Sano T , Kato H , et al. Live imaging of extracellular signal‐regulated kinase and protein kinase A activities during thrombus formation in mice expressing biosensors based on Forster resonance energy transfer. J Thromb Haemost. 2017;15:1487–99. 28453888 10.1111/jth.13723

[81] Magwenzi S , Woodward C , Wraith KS , et al. Oxidized LDL activates blood platelets through CD36/NOX2‐mediated inhibition of the cGMP/protein kinase G signaling cascade. Blood. 2015;125:2693–703. 25710879 10.1182/blood-2014-05-574491 PMC4408294

[82] Roberts W , Magwenzi S , Aburima A , Naseem KM . Thrombospondin‐1 induces platelet activation through CD36‐dependent inhibition of the cAMP/protein kinase A signaling cascade. Blood. 2010;116:4297–306. 20664056 10.1182/blood-2010-01-265561

[83] Senis YA , Mazharian A , Mori J . Src family kinases: at the forefront of platelet activation. Blood. 2014;124:2013–24. 25115887 10.1182/blood-2014-01-453134 PMC4186533

[84] Burkhart JM , Gambaryan S , Watson SP , et al. What can proteomics tell us about platelets? Circ Res. 2014;114:1204–19. 24677239 10.1161/CIRCRESAHA.114.301598

[85] Nagy Z , Senis YA . Ups and downs of the ADP phosphoproteome. Blood. 2017;129:135–6. 28082287 10.1182/blood-2016-11-751917

[86] Senis YA . Protein‐tyrosine phosphatases: a new frontier in platelet signal transduction. J Thromb Haemost. 2013;11:1800–13. 24015866 10.1111/jth.12359

[87] Aslan JE , McCarty OJ . Rho GTPases in platelet function. J Thromb Haemost. 2013;11:35–46. 23121917 10.1111/jth.12051 PMC3928789

[88] Csepanyi‐Komi R , Levay M , Ligeti E . Small G proteins and their regulators in cellular signalling. Mol Cell Endocrinol. 2012;353:10–20. 22108439 10.1016/j.mce.2011.11.005

[89] Goggs R , Williams CM , Mellor H , Poole AW . Platelet Rho GTPases‐a focus on novel players, roles and relationships. Biochem J. 2015;466:431–42. 25748676 10.1042/BJ20141404 PMC4357237

[90] Subramanian H , Zahedi RP , Sickmann A , Walter U , Gambaryan S . Phosphorylation of CalDAG‐GEFI by protein kinase A prevents Rap1b activation. J Thromb Haemost. 2013;11:1574–82. 23611601 10.1111/jth.12271

[91] Guidetti GF , Manganaro D , Consonni A , Canobbio I , Balduini C , Torti M . Phosphorylation of the guanine‐nucleotide‐exchange factor CalDAG‐GEFI by protein kinase A regulates Ca(2+)‐dependent activation of platelet Rap1b GTPase. Biochem J. 2013;453:115–23. 23600630 10.1042/BJ20130131

[92] Hoffmeister M , Riha P , Neumuller O , Danielewski O , Schultess J , Smolenski AP . Cyclic nucleotide‐dependent protein kinases inhibit binding of 14‐3‐3 to the GTPase‐activating protein Rap1GAP2 in platelets. J Biol Chem. 2008;283:2297–306. 18039662 10.1074/jbc.M706825200

[93] Benz PM , Laban H , Zink J , et al. Vasodilator‐stimulated phosphoprotein (VASP)‐dependent and ‐independent pathways regulate thrombin‐induced activation of Rap1b in platelets. Cell Commun Signal. 2016;14:21. 27620165 10.1186/s12964-016-0144-z PMC5020514

[94] Nagy Z , Wynne K , von Kriegsheim A , Gambaryan S , Smolenski A . Cyclic nucleotide‐dependent protein kinases target ARHGAP17 and ARHGEF6 complexes in platelets. J Biol Chem. 2015;290:29974–83. 26507661 10.1074/jbc.M115.678003 PMC4705978

[95] Gratacap MP , Payrastre B , Nieswandt B , Offermanns S . Differential regulation of Rho and Rac through heterotrimeric G‐proteins and cyclic nucleotides. J Biol Chem. 2001;276:47906–13. 11560922 10.1074/jbc.M104442200

[96] Aburima A , Walladbegi K , Wake JD , Naseem KM . cGMP signaling inhibits platelet shape change through regulation of the RhoA‐Rho Kinase‐MLC phosphatase signaling pathway. J Thromb Haemost. 2017;15:1668–78. 28509344 10.1111/jth.13738

[97] Huang Y , Joshi S , Xiang B , et al. Arf6 controls platelet spreading and clot retraction via integrin alphaIIbbeta3 trafficking. Blood. 2016;127:1459–67. 26738539 10.1182/blood-2015-05-648550 PMC4797022

[98] Karim ZA , Choi W , Whiteheart SW . Primary platelet signaling cascades and integrin‐mediated signaling control ADP‐ribosylation factor (Arf) 6‐GTP levels during platelet activation and aggregation. J Biol Chem. 2008;283:11995–2003. 18326492 10.1074/jbc.M800146200 PMC2335347

[99] van den Bosch MT , Poole AW , Hers I . Cytohesin‐2 phosphorylation by protein kinase C relieves the constitutive suppression of platelet dense granule secretion by ADP‐ribosylation factor 6. J Thromb Haemost. 2014;12:726–35. 24581425 10.1111/jth.12542 PMC4238808

[100] Freson K , Hoylaerts MF , Jaeken J , et al. Genetic variation of the extra‐large stimulatory G protein alpha‐subunit leads to Gs hyperfunction in platelets and is a risk factor for bleeding. Thromb Haemost. 2001;86:733–8. 11583302

[101] Emdin CA , Khera AV , Klarin D , et al. Phenotypic consequences of a genetic predisposition to enhanced nitric oxide signaling. Circulation. 2018;137:222–32. 28982690 10.1161/CIRCULATIONAHA.117.028021 PMC5771958

[102] Freson K , Izzi B , Jaeken J , et al. Compound heterozygous mutations in the GNAS gene of a boy with morbid obesity, thyroid‐stimulating hormone resistance, pseudohypoparathyroidism, and a prothrombotic state. J Clin Endocrinol Metab. 2008;93:4844–9. 18796523 10.1210/jc.2008-0233

[103] Malik R , Chauhan G , Traylor M , et al. Multiancestry genome‐wide association study of 520,000 subjects identifies 32 loci associated with stroke and stroke subtypes. Nat Genet. 2018;50:524–37. 29531354 10.1038/s41588-018-0058-3 PMC5968830

[104] Shinohara Y , Katayama Y , Uchiyama S , et al. Cilostazol for prevention of secondary stroke (CSPS 2): an aspirin‐controlled, double‐blind, randomised non‐inferiority trial. Lancet Neurol. 2010;9:959–68. 20833591 10.1016/S1474-4422(10)70198-8

[105] Rothwell PM , Algra A , Chen Z , Diener HC , Norrving B , Mehta Z . Effects of aspirin on risk and severity of early recurrent stroke after transient ischaemic attack and ischaemic stroke: time‐course analysis of randomised trials. Lancet. 2016;388:365–75. 27209146 10.1016/S0140-6736(16)30468-8 PMC5321490

[106] Gresele P , Momi S , Falcinelli E . Anti‐platelet therapy: phosphodiesterase inhibitors. Br J Clin Pharmacol. 2011;72:634–46. 21649691 10.1111/j.1365-2125.2011.04034.x PMC3195739

[107] Munzel T , Daiber A . Inorganic nitrite and nitrate in cardiovascular therapy: a better alternative to organic nitrates as nitric oxide donors? Vascul Pharmacol. 2018;102:1–10. 29174923 10.1016/j.vph.2017.11.003

[108] Ghofrani HA , Humbert M , Langleben D , et al. Riociguat: mode of action and clinical development in pulmonary hypertension. Chest. 2017;151:468–80. 27263466 10.1016/j.chest.2016.05.024

[109] Mega JL , Hochholzer W , Frelinger AL 3rd , et al. Dosing clopidogrel based on CYP2C19 genotype and the effect on platelet reactivity in patients with stable cardiovascular disease. JAMA. 2011;306:2221–8. 22088980 10.1001/jama.2011.1703

[110] Storey RF , Angiolillo DJ , Bonaca MP , et al. Platelet inhibition with ticagrelor 60 mg versus 90 mg twice daily in the PEGASUS‐TIMI 54 trial. J Am Coll Cardiol. 2016;67:1145–54. 26965534 10.1016/j.jacc.2015.12.062

[111] Eikelboom JW , Hirsh J , Spencer FA , Baglin TP , Weitz JI . Antiplatelet drugs: antithrombotic Therapy and Prevention of Thrombosis, 9th ed: American College of Chest Physicians Evidence‐Based Clinical Practice Guidelines. Chest. 2012;141:e89S–119S. 22315278 10.1378/chest.11-2293 PMC3278069

[112] El‐Brolosy MA , Stainier DYR . Genetic compensation: a phenomenon in search of mechanisms. PLoS Genet. 2017;13:e1006780. 28704371 10.1371/journal.pgen.1006780 PMC5509088

[113] El‐Daher SS , Patel Y , Siddiqua A , et al. Distinct localization and function of (1,4,5)IP(3) receptor subtypes and the (1,3,4,5)IP(4) receptor GAP1(IP4BP) in highly purified human platelet membranes. Blood. 2000;95:3412–22. 10828023

[114] Danielewski O , Schultess J , Smolenski A . The NO/cGMP pathway inhibits Rap 1 activation in human platelets via cGMP‐dependent protein kinase I. Thromb Haemost. 2005;93:319–25. 15711749 10.1160/TH04-09-0582

